# Dr. Bradley on an Epidemic Puerperal Fever

**Published:** 1811-03

**Authors:** 


### Medical and Physical Journal.

VOL. XXV.] MARCH, 1811. [NO. 145.

Printed for R. PHILLIPS, by E. Hemsted, Great New Street, Fetter Lane, London.

*To the Editors of the Medical and Physical Journal.*

### *Dr. Bradley on an epidemic Puerperal Fever.*

GENTLEMEN,

IN the west-riding of this county we have had, for more than twelve months past, an epidemic puerperal fever, which has excited such alarm among pregnant females, especially in some of the larger towns, as to induce many to remove to distant places for the period of their confinement. From the best information I can collect, this fever has been exceedingly fatal, insomuch, that for several months after its first appearance, none, I believe, recovered.

I have not yet learnt that any medical man has favoured the public with remarks on this most formidable complaint, in which, I am happy to say, my experience has been very circumscribed; and I am therefore incompetent to offer any pathological information, as the result of extensive practice. I have seen but six of these cases, three of which were in the last stage of the complaint, and only survived a few hours after first seeing them. A fourth, I attended at an earlier period of the disorder, but was not more successful; and the two last (of one of which I can only give a summary detail), and which constitute the subject of this communication, I have sent you for insertion, if you deem them worthy of a place in your Journal. These involve neither much novelty nor interest, but with regard to their therapeutic or medical treatment, they may not only afford some practical hints, but matter of reflection to a few of my provincial brethren.

Mrs. Wood, aged thirty-two years, of a full habit and a sanguine constitution, was eighteen hours after delivery of her first child, on the 6th of December last, seized with a severe rigor, succeeded by nausea, heat, and great pain in the hypogastric region, especially towards the left side. Eleven hours after this attack, I found her complaining of great pain, soreness, and tension of the lower part of the abdomen, insomuch, that she could scarcely bear its being touched. Her pulse was at 122, small, thready, and irregular. She had great thirst, with a tongue a good deal furred, and inclinable to be yellow, and was costive. She made water tolerably well, but the lochia was in very small quantity, and flowed irregularly, and there were no signs of lactation. The pain in her belly was so severe, as to prevent her receiving any rest, and to confine her to one position; for she was the easiest when laid on her back or rather inclining to the right side. Her respiration was also rather obstructed, yet she was without either cough, or pain in the chest, but complained of head-ach.

About three hours previous to my seeing her, she had lost eighteen ounces of blood from the arm, which appeared rather sizy, and somewhat relieved her pain. She had likewise taken opening medicine which had not operated. I ordered her fifteen grains of the hydrarg. subm. in a bolus, to be taken immediately, drinking after it a scruple of jalap, and eight grains of purified nitre, made into a draught, and clysters, consisting of a pint of warm water-gruel, well sweetened with coarse sugar, with the addition of a spoonful or two of castor-oil, to be frequently injected. She was also ordered to dilute well, by drinking large quantities of balm-tea, water-gruel, barley-water, &c.; and her diet to consist of tea, cocoa, and weak chicken-broth. After an interval of six hours, I found the medicine had operated five times, and that her pulse was no quicker, and rather less irregular. Her tongue, also, was in no respects worse, and she declared that every motion had procured her additional relief. Ordered of the hydrarg. subm. and pulv. jalap, each six grains, to be given in the form of a bolus, swallowing after it a draught, consisting of the solution of a dram of the magnes. sulph. and to be repeated every four hours, with the exception of the hydrarg. subm. and jalap being reduced to five grains each dose. Whence, it will be seen, that in the first twenty-four hours she took thirty-three grains of hydrarg. subm. thirty-seven of jalap, and half an ounce of magn. sulph. These, with the occasional administration of clysters, procured about ten copious motions. In the evening her pulse continued the same in number as in the morning, but was less irregular, and more distinct. Her thirst also was diminished, and her tongue no more furred, and was less yellow. The pain and soreness of the abdomen were greatly relieved, and she felt little of them, except on motion or pressure. A considerable circumscribed hardness, however, occupied the hypogastric region, especially towards the left side. This appeared to me to be internal, and on on pressure gave more pain than any of the contiguous parts. Her face was likewise flushed, and though her skin was hot and without moisture, yet her spirits were better, and she slept two hours this evening, which she attributed to a diminution of her pain.

7th. Her pulse this morning was small, and at 120, but softer and less irregular, and she had slept about one third of the night, at intervals. Her tongue was equally furred, but rather whiter, and her thirst not immoderate. She was also free from rigors, but complained of being hot in the night; and her countenance was still a good deal flushed this morning. The abdomen continued to be less sore, and tumefied, and the hardness, already described, was rather diminished. The lochia was still in very small quantities, and sometimes for a while suspended, and the urine, as far as could be ascertained, was high-coloured. In the evening her pulse was soft, less irregular, somewhat more elevated, and at 108; her tongue rather cleaner, and her thirst still moderate. Her urine also was paler, and her skin had been more moist, and somewhat cooler all the day than at any time since her first attack. In other respects no variation. In the last twenty-four hours, she took half a dram of hydrarg. subm. thirty-one grains of jalap, and half an ounce of magnes. sulph. at nearly three equal portions, and at the distance of six hours betwixt each dose. These procured nine or ten liquid stools, most of which were slimy, though copious. Ordered the following liniment to be rubbed gently, and for some time, on the abdomen, twice or three times a day.

R. Lin. Ammon. Carb. ℥x. Camphor. ℥i.

8th. This morning she complained of uneasiness, and some increase of pain, and tumefaction of the abdomen, for about two hours, but by a copious stool, (a discharge that had been interrupted for the last five or six hours) together with a great deal of the crepitus alvinus, and at the same time a small return of the lochia, which had also disappeared, the above symptoms considerably abated. Her pulse was soft, small, and at 106, and her tongue was daily getting cleaner; her thirst also diminishing; her skin becoming moister, and her urine more pale. She slept little yesterday, but in the course of the night had some hours of comfortable repose. The child was ordered to the breasts, as they evidently were distended with milk. She also complained this morning for the first and only time, of her mouth being somewhat sore. In the evening her pulse was at 104, and her belly less sore, painful, and tumefied. The greatest part of her pain was confined to the epigastric region. In

 other other respects, as in the morning. She took this day at eleven in the forenoon, of the hydrarg. subm. and jalap, each ten grains, and two scruples of magn. sulph. These were repeated at five in the afternoon, and at eleven at night, the consequence was eight loose stools, which, she is confident, always relieve, without weakening her.

9th. Her pulse this morning was at 100, small and soft, and her tongue gradually becoming cleaner. She slept tolerably in the night, and was pretty free from thirst. Her skin was moist and temperate as to heat, and her belly also was soft and but little swelled, and without either pain or soreness, except only on considerable pressure. Her urine still continued to grow paler, and her breasts were distended with milk, notwithstanding the lochia occasionally appeared fresh and in small quantities. She took this day of the hydrarg. subm. and jalap, each eight grains night and morning only, which were productive of four liquid, though somewhat mucous stools. She had greater inclination for nutriment, consequently her broths were ordered to be made stronger.

10th. She slept moderately in the night, and was full as well this morning as yesterday. The mammary secretion is now adequate to the support of the child. In the last twenty-four hours she took only six grains of the hydrarg. subm. with the same quantity of jalap, and repeated them but once, at the distance of eight hours. These, however, were sufficient to procure six motions. In the course of the day, she complained of some pain in her bowels, with also some soreness and distention of the abdomen; her pulse, however, was small, soft, and at 96, and her tongue nearly clean; but the lochia, which yesterday was pale and in very small quantity, had disappeared this morning, as I afterwards learnt. Ordered the boluses to be discontinued, and the magnes. sulph. to be substituted, in the dose of half a dram every two hours, or in such a quantity, along with the occasional repetition of her clysters, as to be sufficient to keep her bowels in rather a lax state.

12th. She had a good night, and this morning was free from pain in the bowels, or from tension of the abdomen. Her pulse, also, was soft, and at 95, and her skin moist. She likewise continues taking more nourishment, and sat up to-day and was chearful. Her farther recovery was progressive, and uninterrupted, till the morning of the 15th, when she again was seized with a severe rigor, succeeded by heat, thirst, and a slight erythematous inflammation of the right mamma, which, by fomentations, leeches, and some cooling purgative medicine, disappeared. On the morning, however, however, of the following day, she was suddenly attacked with pain, about the middle of the dorsal vertebræ, which extended through the chest to the cartilago ensiformis, and sometimes shooting under the right mamma. I did not see her after this second attack, till the morning of the 18th, when I found her pain so acute, as not only to obstruct respiration, but to prevent her lying down. Her pulse was at 104, and small, but not irregular, and her tongue was rather furred and yellowish, but her thirst was not immoderate. Her urine also was high coloured, her skin moist, and her bowels were rather lax from the opening medicine she had taken. She had likewise some cough with a slight expectoration, of a whitish mucus. Ordered her to lose eight ounces of blood, and to take the following emetic, as she had some degree of nausea :—R. Pulv. ipecac. ℈j. vin. antim. ʒi. aq. font. ʒiij. M. This operated well, and after it she was directed to take as follows :—R. Pulv. antim. gr. iij. cons. ros. can. q. s. f. bol. quarta quaq. hora sumend. superbibend. cochl. iij. ampl. mist. seq. R. Liq. ammon. acet. ℥ij. spt. ether. nitr. ʒiss. aq. menth. s. ℥ss. aq. font. ℥vss. syr. caryoph. ℥ij. M. In the evening, I found the pain had nearly left her, and her pulse at 94. Her tongue was much the same, but her respiration was freer, her cough less troublesome, and she could bear to lie down in bed, but only on her back, and rather inclining to the left side. Some hours, however, later in the evening, the pain returned, and occupied the right side, about the sixth or seventh rib, to which a blister was ordered to be applied.

19th. Pulse soft at 102, and her tongue was rather more yellow, probably from the blister. She had slept tolerably, and perspired in the night. Her breathing was not much oppressed, but her cough was troublesome, and her expectoration copious and of a brownish colour. On account of having four loose motions in the night the pulv. antim. was discontinued till to-morrow. In other respects no variation since yesterday.

20th. Her pulse this morning was at 98, and her tongue rather cleaner and less yellow, and her thirst inconsiderable. Her left breast was full of milk, but her right breast had been empty ever since the inflammation in it had disappeared. The blister had relieved the pain in her side, and as to other circumstances, much the same as before.

21st. Pulse at 92, and she was costive; for which occasional clysters were only recommended. In other respects little alteration.

22d. This day her pulse was at 96, and she had more thirst. Her urine was hotter and less in quantity. Her respiration, spiration, also, was more difficult, and the pain in her side higher up, and increased; but the cough less troublesome.

23d. She was very ill in the night, but had perspired profusely, which had greatly relieved her. Her pulse this morning was down at 90, and her pain and heat considerably diminished. Her expectoration, also, was more copious, her breathing more free, and her urine paler, with a good deal of sediment.

24th. Her pulse had, this morning, descended to 80, and she was without either thirst or pain. In short, all the febrile symptoms soon left her, and her recovery was speedy and uninterrupted; yet, notwithstanding, her cough continued troublesome, and her expectoration plentiful, for about a week longer, after which, they suddenly disappeared.

---

The other case, of which I promised to give a summary detail, comprehending some particulars not unworthy to be known, was a woman (Mrs. North), who after having many children, was, twelve days after her last accouchement, seized with a violent pain, a little above the spine of the right os ilium, inclining towards the navel. This was accompanied with most of the symptoms peculiar to Mrs. Wood's case, excepting the pulse, which was less quick, though in other respects the same. Her tongue, however, was browner and more furred, but the tension and soreness of the abdomen were less. Her thirst was not quite so immoderate, but her urine was rather higher coloured. The lochia, also, had ceased, and her milk had been dispersed several days before her attack. Her pain was so acute, as to shoot up into different parts of the chest, to obstruct respiration, and to admit scarcely of any relief from the loss of twenty-six ounces of blood at two operations, nor from blistering, nor cooling purgatives. Under these circumstances of distress, I thought the plan of giving calomel and opium, as recommended by Dr. Hamilton, of Lynn-Regis, in pleurisy, and some other inflammatory affections, might here be adopted, with some prospect of relief. She therefore was ordered a grain and a half of the hydrarg. subm. and three-fourths of a grain of the ext. opii, to be taken every six hours, from which she soon experienced a mitigation of her pain; in ten or twelve hours, her pulse, tongue, and other symptoms, assumed appearances of amelioration; and in less than twenty-four hours after first taking this medicine, a slight ptyalism appeared. From this circumstance it was judged proper to discontinue the medicine for a day, when again it was had recourse to with the happiest effects. This was repeated several veral times in the course of the complaint, and from circumstances discontinued. It never affected the mouth but once, though the dose was increased to one third more, and longer persisted in; and besides, it must be acknowledged, that as often as it was resorted to, its good effects were somewhat less distinguishable. The pulse, from the third day to the end of the first fortnight, was frequently vacillating from 100 to 112, and apparently under the influence of local irritation; yet the medicine not only abated the latter, but ameliorated the state of the former, by rendering it softer, more distinct, and less irregular; and also by diminishing the brown and unpleasant appearance of the tongue, and promoting an increased secretion of urine, and perspiration, and rendering the former paler.

In this case of Mrs. North, I was led to suspect that calomel was the principal agent in producing these effects, I therefore determined whenever such a case again occurred to push its administration to a greater extent.

On my first seeing this woman I thought her complaint was a case of peritonitis, as there were not that tension and soreness as in the preceding instance: but I soon was convinced of its being a case of puerperal inflammation, and a variety of the same species of epidemic before alluded to. My reasons for this conclusion were, First, because the pulse here was small, thready, irregular and somewhat creeping, and very similar to that, which, I believe, attends this complaint, when well marked. Secondly, because the tongue exhibited a more typhoid appearance than usually attends peritonitis only. Thirdly, that this case resembled in all its leading features, that of a woman not many weeks antecedent, in the same neighbourhood, who was seized with puerperal inflammation within twenty-four hours subsequent to delivery, and died in less than forty-eight hours after its attack. Fourthly, that the case of Mrs. Wood happening in less than a fortnight afterwards, and in the immediate vicinity, their respective places of residence being not more than seventy yards distance, strongly evince, if taken in conjunction with the preceding circumstances, the true nature of her complaint.

Whatever, on further experience, may be thought of the virtues of calomel, as a specific in this affection, it is evident in this instance of Mrs. Wood in particular, that it arrested the progress of her complaint, a circumstance, I think, that may be fairly ascribed to the quantity administered. On experiencing the good effects of calomel in Mrs. North's treatment, I was determined, if another opportunity offered, to introduce it more liberally into the system, cautiously, however, ever, observing its effects, and regulating my conduct by them.

When I gave Mrs. Wood the first dose, I had not prescribed in my own mind the mode of treatment I should pursue; nevertheless, I was partial to the plan I had adopted in Mrs. North's case; but, when I found that the first purgative dose produced such pleasing effects, and that from every fresh motion she found increased relief, I was led by these circumstances to repeat the large doses, combined as already narrated; and so far were these from inducing any symptoms of debility, that they evidently produced a contrary effect, not only by ameliorating the pulse and tongue, but as it were exciting a new action in the system; for on my first visit, though her skin was hot, yet her countenance was rather pale and dejected, and expressive of that languor and want of vital energy which is partly peculiar to this affection, but in less than twenty-four hours, her whole countenance was flushed, her spirits better, and she was more sanguine of recovery.

She never complained of her medicines operating too much, but more than once observed, that, when her stools were less frequent, she experienced an increased degree of distress. After the operation of the first dose of her medicine the local irritation abated, insomuch as not to return, except in a partial degree. I have not seen similar effects produced in the few instances of this complaint, which I have met with, by any other purgative. Hence, we may presume, it possesses some anodyne properties.

Whether if the calomel had been longer persisted in, the pleuritic symptoms would have made their appearance, is a question, that perhaps will not be easily solved; but the reason why this medicine was discontinued so soon, was, on account of the griping pain in the bowels, and soreness of the abdomen. These circumstances, together with the mammary and other secretions being copious, impressed me with a conviction, that it was no longer necessary, and by being persisted in, might produce fresh symptoms of irritation, not dissimilar to those, which it had so happily relieved. This supposition, perhaps, was erroneous, for a few hours after discontinuing the calomel she had a slight return of the lochia, which had been for twelve hours previously suppressed, and almost immediately after which, all symptoms of irritation vanished.

Some farther particulars, relating to Mrs. North, it may not be improper to relate. About a fortnight after every symptom of fever and local inflammation had disappeared, and after having regained her appetite, and strength, so as to be enabled to go about her household affairs, she was suddenly seized seized with apoplexy, which in a few moments terminated her existence.

It may be necessary to remark, this woman had been exceedingly corpulent many years, had a short neck and was about 35 years of age.

*Huddersfield, Jan.* 10, 1811.

